# Investigating the role of F_O_F_1_ ATPase in *Zymomonas mobilis* through deletion of its F_O_ and F_1_ subcomplexes

**DOI:** 10.1186/s12934-026-03059-x

**Published:** 2026-07-13

**Authors:** Gerrich Behrendt, Reinis Rutkis, Uldis Kalnenieks, Katja Bettenbrock

**Affiliations:** 1 Max-Planck-Institute for Dynamics of Complex Technical Systems, Sandtorstraße 1, 39106 Magdeburg, Germany; 2 Institute of Microbiology and Biotechnology, University of Latvia, 1 Jelgavas Str., Riga, LV-1004 Latvia

**Keywords:** FoF1 ATPase, Uncoupled growth, Respiration, *Zymomonas mobilis*

## Abstract

*Zymomonas mobilis* has a typical F_O_F_1_ ATPase, yet does not exhibit oxidative phosphorylation. In order to investigate the function of the ATPase we sought to delete the F_O_ and the F_1_ subcomplexes, respectively. Clean mutants could be obtained after selection under aerobic conditions. Growth analysis of the mutants showed that they strictly require oxygen for growth and that the oxygen supply is a factor determining maximal growth rate. This is contrary to the wild type, which grows fastest under anaerobic conditions. In the mutant strains, ethanol remains the main catabolic product; however glucose consumption rates are reduced, and the mutants show a higher sensitivity to low medium pH. Compared to the wild type, the mutants show an increased biomass yield. Our data support the previously proposed regulatory role of the F_O_F_1_ ATPase in controlling glycolytic flux, and indicate that the F_O_F_1_ ATPase plays an important role in expelling protons from the cytoplasm. In the mutants, this function is taken over by the electron transport chain in the presence of oxygen.

**Supplementary Information:**

The online version contains supplementary material available at 10.1186/s12934-026-03059-x.

## Introduction

*Zymomonas mobilis* is an ethanologenic alpha-proteobacterium that shows great promise as a chassis for bulk chemical production. It is unique in using solely the Entner–Doudoroff pathway under anaerobic conditions [50] resulting in a far higher ethanol titer, productivity, and yield than other ethanologenic microbes [37].

A notable feature of *Z. mobilis* is its energetically uncoupled growth phenotype [4] meaning that high rates of glucose uptake and ATP production are not reflected in biomass production. While this is a favorable property for production, the underlying mechanisms are unknown. Perhaps, ATP is spilled by so far uncharacterized reaction(s). In this regard, ATP wasting or proton leakage at the membrane could explain the uncoupled growth phenotype [18]. An involvement of the F_O_F_1_ ATPase of *Z. mobilis* is likely [20, 36]. Overproduction of the F_1_ subunit of the ATPase could lead to ATP cleavage that is not coupled to any useful reaction. This type of “ATP wasting” has been exploited in biotechnological applications in order to increase substrate uptake rates [5, 27]. Another possibility is the dissipation of the H^+^ gradient at the membrane due to elevated proton permeability of the cytoplasmic membrane.

The F_O_F_1_ ATPase typically acts in both directions: ATP synthesis and ATP cleavage. Both processes are coupled to the movement of protons across the membrane. During oxidative phosphorylation under respiratory conditions, the proton gradient established by the electron transport chain (ETC) can be used for the production of ATP coupled with an inflow of protons. Under anaerobic conditions, protons can be actively pumped out of the cell, driven by ATP cleavage, in order to maintain the proton motive force at the membrane [46].

*Z. mobilis* has a typical F_O_F_1_ ATPase [36]. However, contrary to most bacteria, where the ATP synthase encoding genes are organized in a single transcription unit (*atpIBEFHAGDC*) [15], *Z. mobilis* has two separate loci that encode the proteins of the F_O_ (ZMO2005, ZMO0667-0671, *atpI-atpB-atpE-atpG2-atpF*) and F_1_ (ZMO0238-0241, *atpH-atpA-atpG1-atpD-atpC*) subcomplexes of the ATP synthase, respectively [43]. This organization is similar to that found in cyanobacteria [9, 30, 38]. Transcription of F_O_ and F_1_ from independent loci would, in principle, permit non-stoichiometric expression and independent regulation of the two sectors. This property is exploited in cyanobacteria, where the two operons respond differentially to environmental and metabolic cues [31, 39]. Whether *Z. mobilis* makes use of this regulatory flexibility, for example, to tune F_1_:F_O_ stoichiometry under conditions where the ATPase operates predominantly as a proton pump, remains to be tested. Additionally, unlike other facultative anaerobic bacteria, *Z. mobilis* cultures grown aerobically do not show signs of energy generation via oxidative phosphorylation [4, 7, 22].

*Z. mobilis* is a facultative anaerobic bacterium equipped with an ETC consisting of a type II NADH dehydrogenase, ubiquinone and a terminal oxidase *bd* that is able to contribute to the buildup of proton motive force, albeit with a low H^+^/e^-^ efficiency [23, 44, 49]. Despite the presence of the ETC and measurable oxygen consumption, *Z. mobilis* exhibits a fermentative metabolism even under aerobic conditions and aerobic growth does not result in increased growth rates or biomass yields [4, 7, 16, 21]. There are conflicting reports on whether growth is slightly enhanced or reduced under aerobic conditions. This may depend on the production of acetaldehyde which inhibits cell growth when it accumulates, therefore it is influenced by the specific culturing conditions. In principle however, ATP production by the ATPase of *Z. mobilis* is possible, as demonstrated for starved cells [22] yet the reason why oxidative phosphorylation does not occur during growth remains unknown.

This article investigates the effects of deleting the F_O_ and the F_1_ subcomplexes in *Z. mobilis* ZM4. We showed that the ATPase mutants lose the ability to grow anaerobically. In the presence of oxygen, the mutants exhibit slightly slower growth rates than the wild type, and significantly slower specific rates of glucose uptake and ethanol production. Notably, biomass yields of the mutants are increased. The O_2_ dependency of the mutants suggests that ATPase plays a role in establishing or maintaining the proton gradient at the membrane. In the presence of oxygen, this function can be performed by the electron transport chain.

## Materials and methods

### Strains and media

For plasmid construction *E. coli* NEB5α (NEB) was used. Cultivations were done in LB_0_ medium (10 g/l tryptone, 5 g/l yeast extract, 5 g/l NaCl) at 37 °C with shaking. For work with *Z. mobilis* ATCC 31821 (ZM4) was used as a reference wild-type strain. Cultivations were performed at 30 °C in ZM medium (2 g/l glucose, 5 g/l yeast extract, 1 g/l (NH_4_)_2_SO_4_, 1 g/l KH_2_PO_4_, 0.5 g/l MgSO_4_). Kanamycin and spectinomycin were both used at concentrations of 100 µg/ml. For solid medium 15 g/l of bacto agar was added.

### Generation of F_O_ and F_1_ knock-outs

Knock out of the genes encoding the F_O_ and F_1_subcomplex of the ATPase, respectively, was performed using homologous recombination. Suicide vectors for homologous recombination were assembled based on the Zymo-Parts toolbox [2] with Golden Gate cloning [10]. Homology arms were amplified from wild-type DNA of ZM4 by PCR using Q5 DNA Polymerase (NEB) according to the manufacturer’s recommendations. pZP337 was used to generate ΔF_O_ (ZM4 ΔZMO2005-ZMO0067-ZMO0068-ZMO0069-ZMO0071:: Kan^R^) and pZP338 was used to generate ΔF_1_ (ZM4 ΔZMO0238-ZMO0239-ZMO0240-ZMO0241-ZMO0242 :: Spec^R^). pZP337 gets its homology arms from pZP064 and pZP065, which carry amplificates generated by primer pairs 138 + 139 for the downstream homology and 140 + 141, 142 + 143 and 144 + 145 for the upstream homology, respectively. Amplification of the upstream homology arm in three fragments was necessary to remove restriction enzyme recognition sites. pZP338 gets its homology arms from pZP068 and pZP069, which carry amplificates generated by primers 148 + 149 for the upstream homology arm and 150 + 151 for the downstream homology arm, respectively. All PCR products were generated from gDNA of ZM4. For the corresponding primers and detailed assembly instructions, see Supplementary Table 1. Plasmid sequences and the sequences of the edited loci can be found as .gb files in the corresponding Edmond repository (10.17617/3.X1LFHY). Suicide vectors were transformed into *Z. mobilis* by electroporation [2]. After selection of kanamycin or spectinomycin resistant colonies, colonies were checked for deletion of the respective genes using PCR. In some cases, heterogeneous colonies were obtained, that showed PCR bands corresponding to the wild-type gene as well as to the knock-out allele. Such colonies were repeatedly inoculated for a few days in selective medium under aerobic conditions to obtain homogeneous knock-out strains.

### Growth analysis

For experiments with varying aeration, *Z. mobilis* cells were grown in 100 ml shake flasks with baffles filled with 20 mL culture in ZM medium. The flasks were incubated either standing on the desktop or with shaking at 50 rpm or 250 rpm, respectively (KS 15, Edmund Bühler GmbH). Anaerobic cultivations were performed in shake flasks, too, but incubated without agitation in an anaerobic workbench. If indicated, KCN was added to a concentration of 100 µM.

For bioreactor experiments an Infors Multifors system with 1.5 L cultivation vessels was used. The vessels were filled with 500 ml of culture. The reactors were stirred at 250 rpm. Aeration was set to 0.5 L min^-1^ using a gas mix of 10% air and 90% N_2_. The pH was adjusted to pH 6.0 using 1 M NaOH. The reactor was connected to BlueVary Off-gas analyzer (BlueSens) for measurement of CO_2_ and O_2_ in the outlet stream.

For continuous cultivation, the pO_2_ was set to 40% and controlled by variations in stirrer speed and air flow. Following an initial batch phase, fresh ZM medium containing 2% glucose was fed continuously to the bioreactor, while the same volume of fermentation broth was withdrawn. The dilution rates were varied from 0.05 h^-1^ to 0.125 h^-1^, corresponding to feed volumes ranging from 25 mL h^-1^ to 62.5 mL h^-1^. The reported values are derived from observations of constant biomass and off-gas values for at least five residence times.

OD_600_ values were converted to biomass in grams by applying a conversion factor of 0.34 g/L at an OD_600_ of 1. To determine this conversion factor, the cells were harvested from the bioreactor by centrifugation and washed twice with water. The pellet was dried at 105 °C for at least 48 h before the dry cell mass was weighed.

Growth rates were determined by plotting the OD_600_ values from the exponential phase against time and applying an exponential fit. Glucose, ethanol and acetaldehyde concentrations were determined by using the D-Glucose HK assay Kit, the Ethanol Assay Kit, and the Acetaldehyde Assay Kit, all from Megazyme. Yields were determined by plotting the respective values against each other and applying a linear regression. Glucose consumption and ethanol production rates were calculated by plotting the glucose or ethanol concentrations against the corresponding biomass concentrations and applying linear regression. The slope was multiplied by the growth rate to obtain the glucose consumption and ethanol production rates. For continuous cultivations glucose consumption rates were determined by calculating the difference between the glucose concentration of the feed solution and the residual concentration in the bioreactor. This value was then divided by the biomass concentration at the respective time point and multiplied by the dilution rate.

CO_2_ production and O_2_ consumption were calculated accordingly using the values from the exhaust gas recorded by the BlueVary exhaust gas analyzer.

### Determination of intracellular pH and pH sensitivity

To test whether the mutants exhibit a different sensitivity to low medium pH, cultivations were carried out in 96-well microtiter plates. A preculture was grown in ZM medium at pH 5.8. Cells were harvested from exponential growth phase by centrifugation and afterwards resuspended to an OD_600_ of ~ 0.25 using ZM medium adjusted to varying pH by addition of HCl. 100 µl of this culture were transferred to each well in 96 well plates. Cultivations were performed using a microplate reader (Vantastar, BMG Labtec) at 30 °C and at a shaking speed of 800 rpm.

The intracellular pH (pH_i_) was investigated using the intracellular pH sensor PHP [1, 32]. The gene encoding PHP was amplified from pS2513-PHP [1] and expressed from a pZMOB06-based shuttle vector with a chloramphenicol selection cassette and the strong promoter P_strong100k*_ together with rbs10k [3]. The plasmid was assembled using the Zymo-Parts toolbox [2] and introduced into the strains via conjugation with ST18 [51]. The cultivation was carried out in ZM media at 30 °C in 24-well plates shaken at 200 rpm in a VantaStar. The ratio of fluorescence emission at λ ~ 515 nm after excitation at λ 405 nm and λ 485 nm, respectively (R405/485) was used to infer the pH_i_ as described for *Pseudomonas* [1].

### Membrane fractions preparation and NADH oxidase : Q1 assay

For assaying NADH: Q1 oxidoreductase activity, cytoplasmic membrane fractions were prepared as described earlier [48]. In short, cells were cultured aerobically for 8 h at 30 °C until reaching the late exponential phase. Cytoplasmic membrane fractions were prepared by harvesting the cells, resuspending them in 0.1 M potassium phosphate buffer (5 mM MgSO_4_, pH 7), and performing ultrasonic breakage by sonication in UP-200s ultrasonic processor (Dr. Hielscher, Germany) for 5 min with pulses of 0.5 s duration, separated by 0.5 s intervals, while maintaining the sample in an ice bath at 0 °C. Unbroken cells were removed, and the membrane fraction was isolated via ultracentrifugation (35,000 rpm for 1 h), followed by repeated washing. The final membrane suspension was prepared in the same buffer, yielding a protein concentration of 3–4 mg mL^-1^. NADH oxidase activity was measured by monitoring the decrease in NADH absorbance at 340 nm (Δε of 6.22 mM^-1^ cm ^- 1^). The assay mixture consisted of 980 µL of 0.1 M phosphate buffer (pH 7), 10 µL of 10 mM NADH, and 10 µL of the membrane fractions suspension. NADH: Q1 oxidoreductase activity was measured by monitoring the decrease in absorbance at 276 nm, corresponding to the reduction of oxidized ubiquinone (Q1) to its reduced form (Q1H_2_, ubiquinol), (Δε of 14.3 mM^-1^cm ^- 1^). The assay mixture consisted of 948 µL of 0.1 M phosphate buffer (pH 7), 15 µL of 10 mM NADH, 10 µL of the membrane fractions suspension, 20 µL KCN (1 M), 7 µL Q1 (1mM).

### Determination of ATPase activity

To assess whether the mutant strains had lost ATP synthase activity mediated by the membrane H^+^-dependent ATPase, while still retaining the potential for ATP generation through the Entner–Doudoroff pathway, starved cells were subjected to acid and glucose pulse experiments as previously described [40]. In short, cells were harvested from the late exponential growth phase by centrifugation and resuspended in 100 mM potassium phosphate buffer (pH 6.9) supplemented with 2 mM magnesium sulfate, to a final biomass concentration of 6.8–7.0 mg dry weight mL^− 1^. The cell suspensions were then incubated at 30 °C for 3 h to induce starvation. Artificial transmembrane pH gradients of 3.5–4.0 units were generated by the addition of 50 µL of 0.1 M HCl to 10 mL of starved cell suspension (3.8–4.0 mg dry weight mL^− 1^ ) prepared in 10 mM phosphate buffer (pH 6.8). For substrate pulse experiments, glucose (50% w/v) was added to a final concentration of 5 mM.

ATPase activity in membrane fractions was determined using cytoplasmic membrane fractions (CMF) prepared according to the method detailed in Sect. "Membrane fractions preparation and NADH oxidase : Q1 assay". The CMFs were resuspended in Tris-Cl buffer containing 10 mM MgCl_2_ (pH of 7.7), to achieve a final protein concentration of 3 to 4 mg dry weight mL^-1^. The assay was performed at 30^°^ C. The ATPase activity was quantified by measuring the rate of ATP decay that results from ATP hydrolysis. This reaction was initiated by adding ATP to a final concentration of approximately 500 µM to cytoplasmic membrane fractions (CMFs) suspension. Samples were subsequently collected and fixed at 10-minute intervals over a 40-minute period to monitor this release. To confirm that the observed activity was attributable to the F_O_F_1_ ATPase, a control experiment was performed. Prior to the assay, *Z. mobilis* ZM4 samples were incubated for 0.5 h with the F_O_ inhibitor N, N-Dicyclohexylcarbodiimide (DCCD) at a concentration of 100 µM, that is twofold higher than the reported inhibitory concentration for the *Z. mobilis* ATPase [40], ensuring effective inhibition of the F_O_ proton channel.

For the measurement of ATP, the Roche ATP Bioluminescence Assay Kit CLS II was employed using the standard luciferin–luciferase method on a TECAN Infinite 200 Pro microplate reader. ATPase activity was quantified by calculating decay of ATP luminescence. The reaction was initiated by adding ATP (500 µM final concentration) to CMFs resuspended in a Tris-Cl buffer containing 10 mM MgCl₂ at pH 7.7.

### Measurement of glucose consumption in non-growing *Z. mobilis* suspension

For preparation of non-growing *Z. mobilis* suspensions, cells were harvested at late exponential growth phase, sedimented via centrifugation at 5000 rpm for 15 min, washed, and resuspended in 100 mM potassium phosphate buffer (pH 6.9) containing 2 mM magnesium sulfate, to a biomass concentration of 2.0 g DCW L^-1^. Glucose consumption experiments were conducted in shaken flasks at 30 °C at 180 rpm, using an initial glucose concentration of approximately 3% (160 mM). Glucose samples were collected every 30 min over a 4 h period and their concentration was measured using HPLC (Agilent 1100 series), equipped with a Biorad Aminex HPX-87 H column.

## Results and discussion

### Construction and selection of F_O_ and F_1_ knock-out mutants

**Fig. 1:**
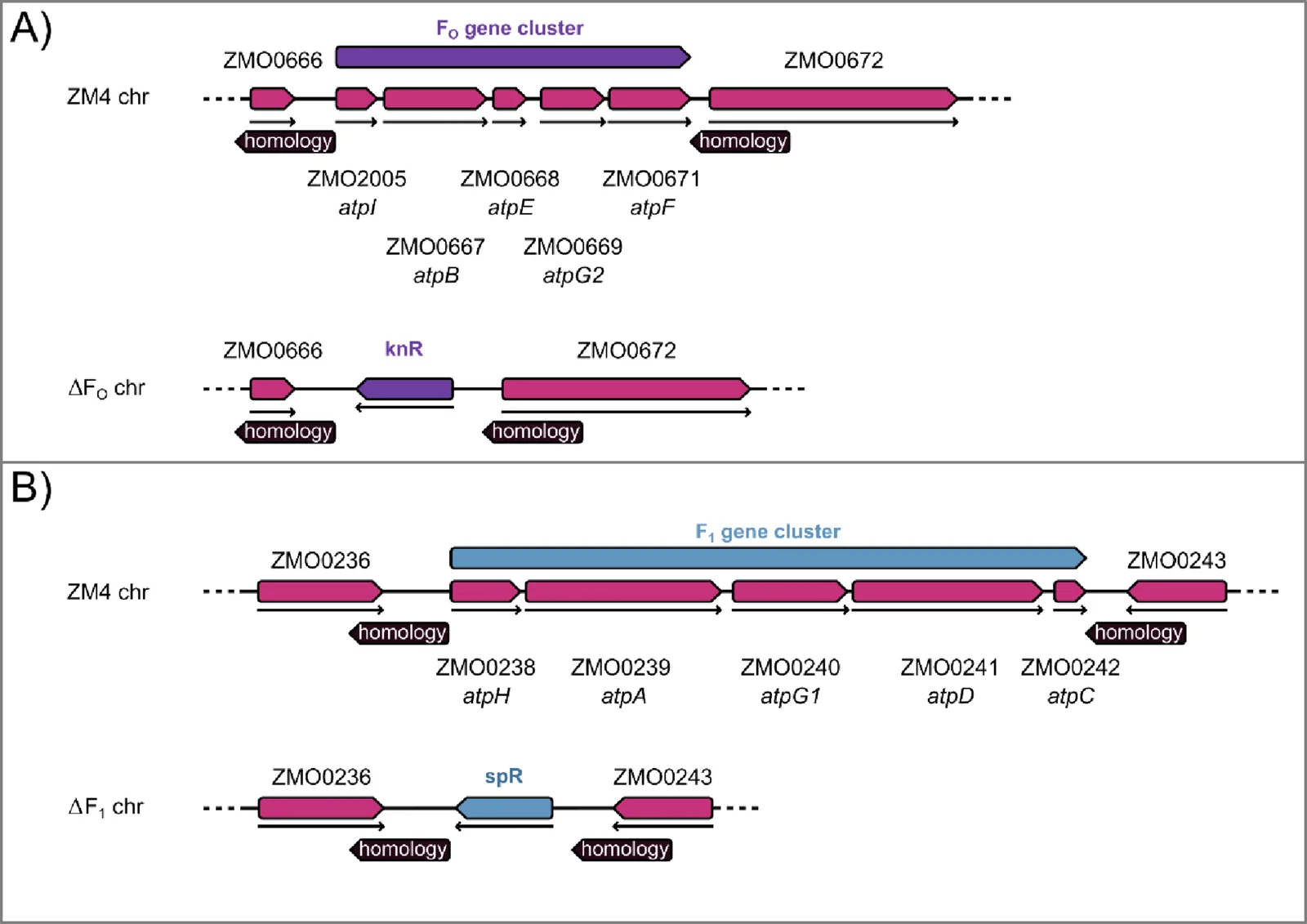
Graphical overview of the gene clusters encoding the **A)** F_O_ and **B)** F_1_ ATPase subcomplexes on the chromosome of *Z. mobilis* ZM4. The region between the homology arms (black) is deleted in the mutants. Below each gene cluster is a map showing the altered chromosomal region in the ΔF_O_ or ΔF_1_ mutant, respectively. Genes are shown with their ZM4 numerical identifiers and coding sequence orientation is indicated with arrows. Intergenic regions surrounding both subcomplex clusters were left intact.

To investigate the function of the F_O_F_1_ ATPase in *Z. mobilis*, we generated mutant strains in which either the genes encoding the F_O_ or the F_1_ subunit of ATPase were deleted using homologous recombination. The deletions only covered the coding regions of the respective genes to minimize the impact on neighboring genes. 800 bp homology arms were selected upstream and downstream of each gene cluster (Fig. 1). Using the Zymo-Parts toolbox [2] suicide vectors were assembled for homologous recombination with a kanamycin and a spectinomycin resistance cassette to replace the F_O_ and F_1_ gene clusters, respectively. After electroporation of the respective suicide vector into the wild-type strain ZM4, several colonies were obtained on plates containing the respective antibiotic. Colony PCR performed to check for either the mutated or the wild-type locus gave positive results for both alleles in all colonies, indicating heterogeneous strains. Because *Z. mobilis* is polyploid, heterogeneous genotypes are possible [6, 13, 14]. Partial knockouts of genes and *Z. mobilis* strains carrying both the knockout allele and the wild-type allele have been described for several genes most notably *pdc* (ZMO1360) [42]. Gene conversion is a mechanism that causes all alleles of a gene to be identical in one cell unless there is selection for both variants [28]. Therefore, we speculated that there had to be a positive selection for the wild-type ATPase under the applied conditions.

In *E. coli* the F_O_F_1_ ATPase is responsible for maintaining the proton gradient across the cytoplasmic membrane under anaerobic conditions [47]. Proton export and maintenance of the proton motive force are also possible through electron transport chains in the presence of suitable electron acceptors. *Z. mobilis* has an active ETC that can use oxygen as an electron acceptor. Therefore, we hypothesized that the positive selection for the wild-type ATPase genes could be circumvented by culturing the mutants under aerobic conditions. After repeatedly inoculating both strains under aerobic conditions in the presence of the respective antibiotic, we obtained homogeneous knock-out strains for ΔF_O_ (ZM4 ΔZMO2005-ZMO0067-ZMO0068-ZMO0069-ZMO0071 :: Kan^R^) and for ΔF_1_ (ZM4 ΔZMO0238-ZMO0239-ZMO0240-ZMO0241-ZMO0242 :: Spec^R^). According to the theory of gene conversion [17, 29, 45], heterogeneous genotypes can only exist if there is selective pressure for the maintenance of both genotypes. In our case, the selection for the maintenance of the WT allele is eliminated by aerobic incubation, allowing antibiotic selection to take effect and leading to allele segregation. Both mutant strains were confirmed by genome sequencing (Suppl Fig. 1). This successful selection strategy for clean F_O_ and F_1_ knockout strains supports the hypothesis that the F_O_F_1_ ATPase is essential for growth under anaerobic conditions in *Z. mobilis*. This finding is consistent with the results from a genome wide CRISPRi screen [11]. As with anaerobically growing *E. coli*, the ATPase may also be important for maintaining the proton gradient across the cell membrane in *Z. mobilis*.

Attempts to delete the genes for both ATPase subcomplexes, and to complement the deletion strains with the genes encoding the respective ATPase subcomplex, were unsuccessful.

### Analysis of the growth behavior of ΔF_O_ and ΔF_1_ mutants

To analyze the growth behavior of the mutants in more detail, the mutants and the parent strain were grown in ZM complex medium with glucose, at different shaking speeds to vary the oxygen supply. Under strictly anaerobic conditions, neither the ΔF_O_ nor the ΔF_1_ mutant was able to grow on plate (Suppl Fig. 2) or liquid culture. However, ZM4 showed a growth rate comparable to that of the standing culture (data not shown). As shown in Table 1, increasing the shaking speed increased the growth rate of the mutants. The shaking speed had less impact on the growth of the ZM4 wild-type strain. Contrary to the mutants, ZM4 exhibited higher growth rates under anaerobic conditions or at slow shaking speed. These results suggest that the mutants require oxygen for growth and that the low dissolved oxygen concentration in standing or slowly shaken cultures prevents them from reaching their maximum growth rate. The mutants therefore benefit from improved oxygen transfer and growth rate appears to depend on oxygen uptake rate. The growth behavior further confirms our assumption that an important function of the ATPase in *Z. mobilis* is establishing a proton gradient at the membrane. If the ATPase fails, the respiratory chain can partially take over this function in the presence of oxygen. Following this logic, inhibiting the respiratory chain would prevent the aerobic growth of the mutants, but not the wild type.

**Table 1:**
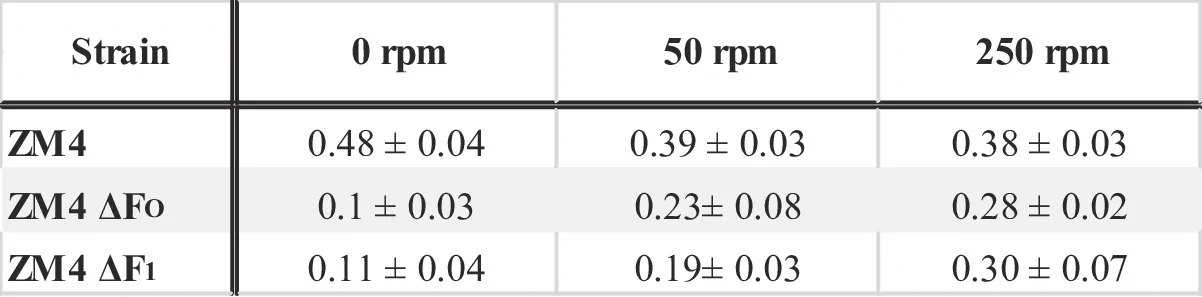
Growth rates of ZM4 and the ATPase mutant strains growing in shake flasks with varying oxygen supplies

Shown are the maximal growth rates [h^− 1^] observed in the growth experiments. The values represent the mean and standard deviations of at least three independent repeats.

**Fig. 2:**
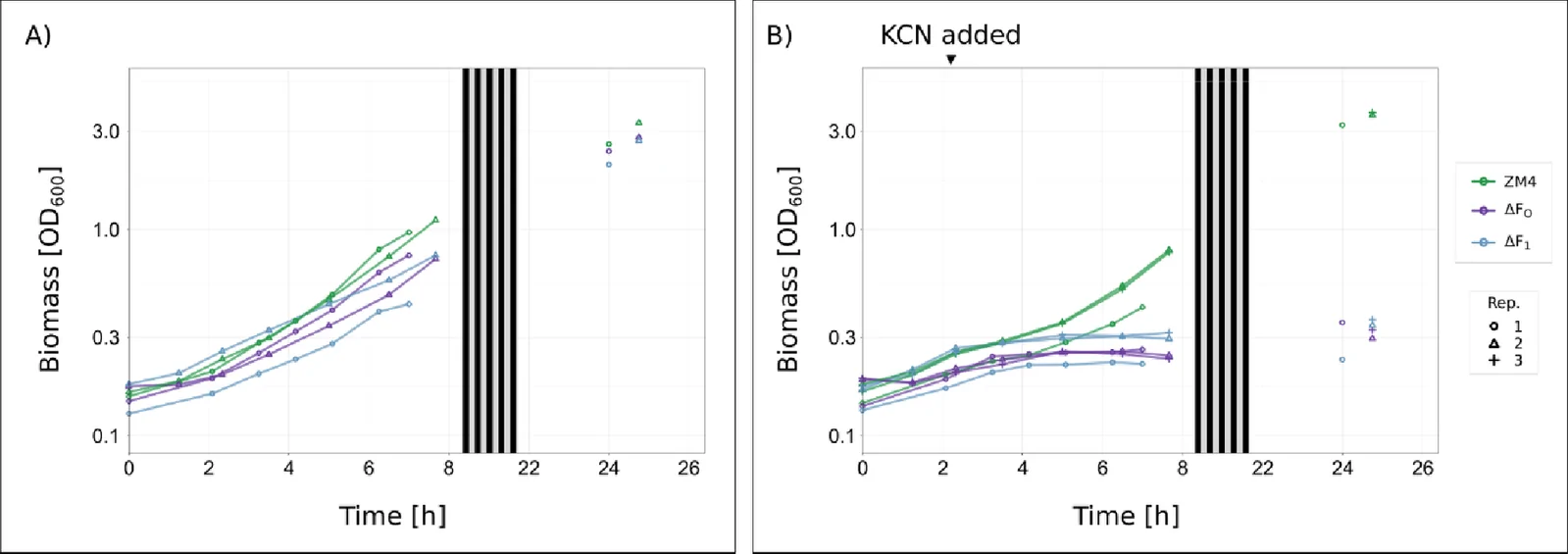
Growth of ZM4, ZM4 ΔF_O_ and ZM4 ΔF_1_ in ZMG in shake flasks **A)** without and **B)** with addition of 100µM KCN. KCN was added after about 2 h incubation when all cultures showed exponential growth. Shown are three independent biological repeats.

Although, as demonstrated by [44], the only terminal oxidase of *Z. mobilis* is structurally homologous to the cyanide-insensitive bd-type oxidase of P. *aeruginosa*, submillimolar potassium cyanide concentrations rapidly inhibit whole cell respiration, and more gradually, also NADH oxidase activity in cytoplasmic membrane preparations [24]. If our hypothesis is correct, the growth of ATPase mutant strains depends on the respiratory chain to establish the membrane proton gradient and therefore requires a functional cytochrome bd oxidase. Therefore, KCN should inhibit growth of the mutants. We added KCN to growing cultures of ZM4 and the mutant strains (Fig. 2). While the addition of KCN had almost no negative impact on the growth behavior of ZM4, both mutants stopped growing in its presence. Previous studies have reported resistance and even a stimulation of growth of ZM6 by KCN, [24]. The fact that KCN inhibits the growth of both mutant strains indicates their dependency on oxygen and the obligatory use of the respiratory chain.

Another small but reproducible difference between the wild-type and the mutant strains was that the wild-type always grew to a higher cell density (OD_600_ ~2.8–3.0) than the mutant strains (OD_600_ ~1.8 -2.0) in shake flasks. This was due to some residual glucose remaining in the cultures of the mutant strains after growth ceased. Since the pH drops during batch experiments with ZM medium to a pH of around 4, the low pH at a later stage of batch growth may explain this behavior.

### Assessment of mutant growth parameters through controlled bioreactor cultivations

Bioreactor experiments enable control of pH during cultivation. They also allow for controlled aeration and off-gas analysis. This led us to perform batch cultivations in bioreactors in order to characterize the mutant strains more thoroughly. The cultures were sparged at a high rate (1 vvm) but the inlet gas consisted of 10% air and 90% N_2_ to restrict the amount of oxygen. This was done to achieve an oxygen supply that was sufficient for good growth of the mutants, while allowing for a good quantification of product formation. Therefore, it is desirable to prevent the production of the volatile product acetaldehyde, which is difficult to quantify. Since we observed low acetaldehyde production when sparging with a mix of 10% air and 90% N_2_, we applied this condition. The high gas flow rate of 1 vvm was chosen, motivated by the conditions used in previous experiments [21]. Table 2 provides an overview of the strains´ performance.

**Table 2:**
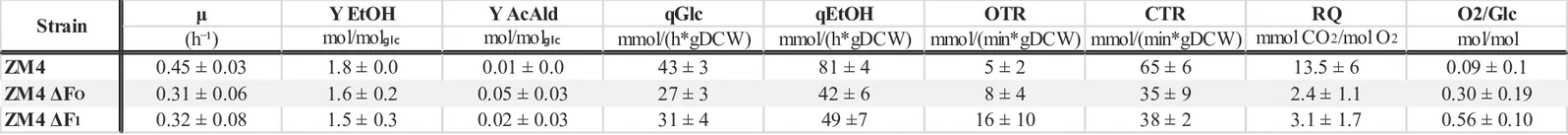
Important growth characteristics of ZM4 and the ΔF_O_ and ΔF_1_ mutants in bioreactor experiments sparged with 10% air + 90% N_2_

Y EtOH is the yield of ethanol produced per mole of glucose. qGlc, qEtOH, OTR, and CTR are the consumption or production rates of the respective substances, calculated per gram of dry cell weight per hour

As can be seen from Table 2 all strains exhibited higher growth rates than those observed in the shake flask experiments (Table 1). This is most likely due to the constant pH of 6.0 and the optimized aeration which provided an adequate but not excessive oxygen supply. Additionally, all strains consumed glucose completely. Under these conditions, the ΔF_1_mutant had the lowest growth rate, although the difference to the growth rate of the ΔF_O_mutant was not significant. Based on experiments with other bacteria (e.g. *E. coli*), we would have expected more pronounced growth defects in the ΔF_O_mutant, because the F_1_subcomplex of the ATPase can cleave ATP when expressed without the F_O_complex. Uncontrolled ATP cleavage by the F1 subcomplex could lead to growth defects, or, if less severe, an increased glucose uptake rate as the cells try to compensate for the ATP loss with a higher glycolytic rate. In *E. coli* overexpressing the F_1_subcomplex leads to ATP cleavage, resulting in higher glycolytic rates and a lower intracellular ATP/ADP ratio [26, 47]. However, separate expression of the F_1_subcomplex did not appear to cause serious problems for *Z. mobilis* energy metabolism, as evidenced by the good growth of the ΔF_O_mutant compared to the ΔF_1_mutant. Consistent with the lower glucose consumption rate we observed lower ethanol production rates for the mutant strains. The mutants also exhibited reduced ethanol yield in comparison to ZM4. However, it is unclear whether this reduction can be considered significant, as the p-values are 0.08 and 0.06, respectively. A reduced ethanol yield would be consistent with our finding that the respiratory chain is required for growth in the mutants. Upon the transfer of electrons from NADH to the respiratory chain, the utilization of NADH for the purpose of ethanol production becomes impeded. Consequently, a decline in ethanol yield is to be anticipated. If the amount of NADH that is not consumed in ethanol synthesis is calculated based on the ethanol yields given in Table 2, the resulting values are on the same order of magnitude as the measured O_2_uptake rates. However, the data, especially the exhaust gas values, are too noisy to completely close the balance. Overall, our data suggest that, in addition to the transmembrane proton transport, ATP cleavage per se is an important function of the F_O_F_1_ATPase in *Z. mobilis*. As previously demonstrated [40], in aerobically growing *Z. mobilis*, not performing oxidative phosphorylation, the F_O_F_1_ATPase functions in the direction of ATP hydrolysis. This potentially contributes to the high glucose consumption rates observed. Although all strains consumed glucose completely, the specific glucose consumption rates (q_Glc_) were reduced by up to 40% in both mutant strains (Table 2). Notably, the behavior of the *Z. mobilis* ATPase mutants contrasts sharply with previous observations on ATPase-deficient *E. coli* [33] and *C.* *glutamicum* [25] strains. In these strains inactivation of the F_O_F_1_ATPase strongly accelerates glycolysis, apparently to compensate for the disruption of oxidative phosphorylation.

The reduction in glycolytic rate is consistent with the principles of Metabolic Control Analysis (MCA), which suggest that control is shared across the pathway rather than residing in single “rate-limiting step.” Specifically, in the kinetic model of the Entner-Doudoroff (ED) pathway in *Z. mobilis*, ATP-consuming reactions exert a high degree of “control” over the glycolytic flux. [41]. Given that the F_O_F_1_ ATPase contribution is estimated to be approximately 20% of the overall ATP turnover in the wild-type *Z. mobilis* [36], according to MCA principles, this decrease in demand (ATP hydrolysis) leads to a proportional decrease in supply (glycolytic flux), thereby reducing the overall rate of glucose consumption. Furthermore, during aerobic growth, a significant proportion of ATP is consumed by energy-intensive processes such as protein synthesis, which can partially mask the ATPase’s contribution to overall ATP consumption. We hypothesized that the regulatory effect of ATPase inactivation would be even more pronounced under non-growing conditions where growth-associated ATP turnover is minimized. To test this hypothesis, we measured the specific glucose uptake rate qGlc in non-growing *Z. mobilis* suspensions.

**Table 3:**
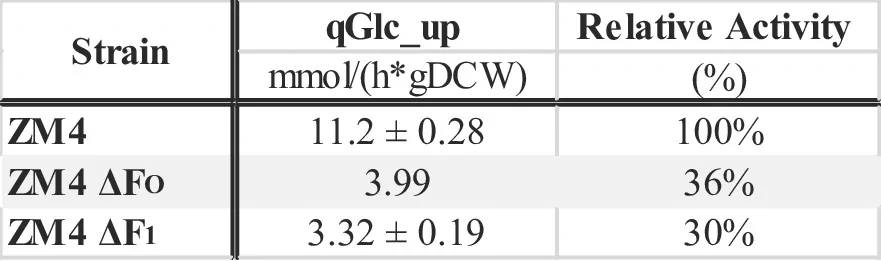
Glucose uptake rates in non-growing *Z. mobilis* ZM4 and ΔF_O_ and ΔF_1_ mutant suspension cultures

Indeed, the differences in qGlc between the wild-type and the mutant strains were more pronounced under non-growing conditions (Table 3). In the mutant strains, the reduction of qGlc was up to 60%. These findings reinforce the premise that F_O_F_1_ ATPase activity contributes significantly to the high glycolytic rate in *Z. mobilis*, although the quantitative relation between the ATPase activity and the glycolytic rate in both mutants requires further study. From a practical perspective, these results suggest modulating the ATP consumption by the F_O_F_1_ ATPase as a viable strategy to vary the glycolytic flux rate in *Z. mobilis*.

Both mutants showed significantly lower glucose uptake and ethanol production rates than the wild-type. (Table 2). Metabolism in the mutants appears to be slowed down and the ethanol yield is reduced. Data from the off-gas analysis (Table 2) indicate that the mutants use higher amounts of oxygen per mole of glucose (0.3 or 0.56 mol_O2_/mol_Glc_) than the wild type (0.09 mol_O2_/mol_Glc_), although again the data are not statistically significant. This is consistent with the idea that the mutants use the respiratory chain to generate the proton motive force. However, the control of the oxygen consumption rate in *Z. mobilis* remains unknown. Despite the elevated oxygen uptake in the mutants, measurements of the NADH:Q1 oxidoreductase activity in the membrane fractions of the parent strain and both ATPase-deficient strains revealed no significant differences (see Supplementary Fig. 3). That may indicate that *Z. mobilis* has a high respiratory chain capacity, which allows an increase in oxygen consumption in the ATPase mutant strains without the need for additional expression of respiratory chain components.

### Analysis of biomass yield in glucose-limited chemostat cultivations

Literature [20, 36] as well as the data shown above indicate that ATP is wasted in *Z. mobilis* in order to maintain a high glucose uptake rate. Significant wasting of ATP, however, should result in reduced biomass yields. Although in some of the batch experiments a slightly higher biomass yield was observed for the F_O_ and F_1_ mutants, the data were very noisy and seemed to depend strongly on aeration and culture conditions (data not shown). To determine the biomass yields of ZM4 and its ΔF_O_ mutant under controlled conditions, we performed a series of continuous cultivations with varying dilution rates from 0.05 to 0.125 h^− 1^. Higher OD_600_ values, corresponding to higher biomass, were observed for the ΔF_O_ mutant compared to ZM4 at all tested dilution rates (Table 4). The average biomass yield in the chemostat cultivations was 0.0372 ± 0.003 g of biomass per gram of glucose for ZM4, compared to 0.0474 ± 0.004 for the ΔF_O_ mutant. This corresponds to an approximately 25% increase in biomass yield. Notably, the yield was slightly lower than the yield calculated for the batch cultivations in Table 2. This could be due to the glucose limitation in the chemostat.

**Table 4:**
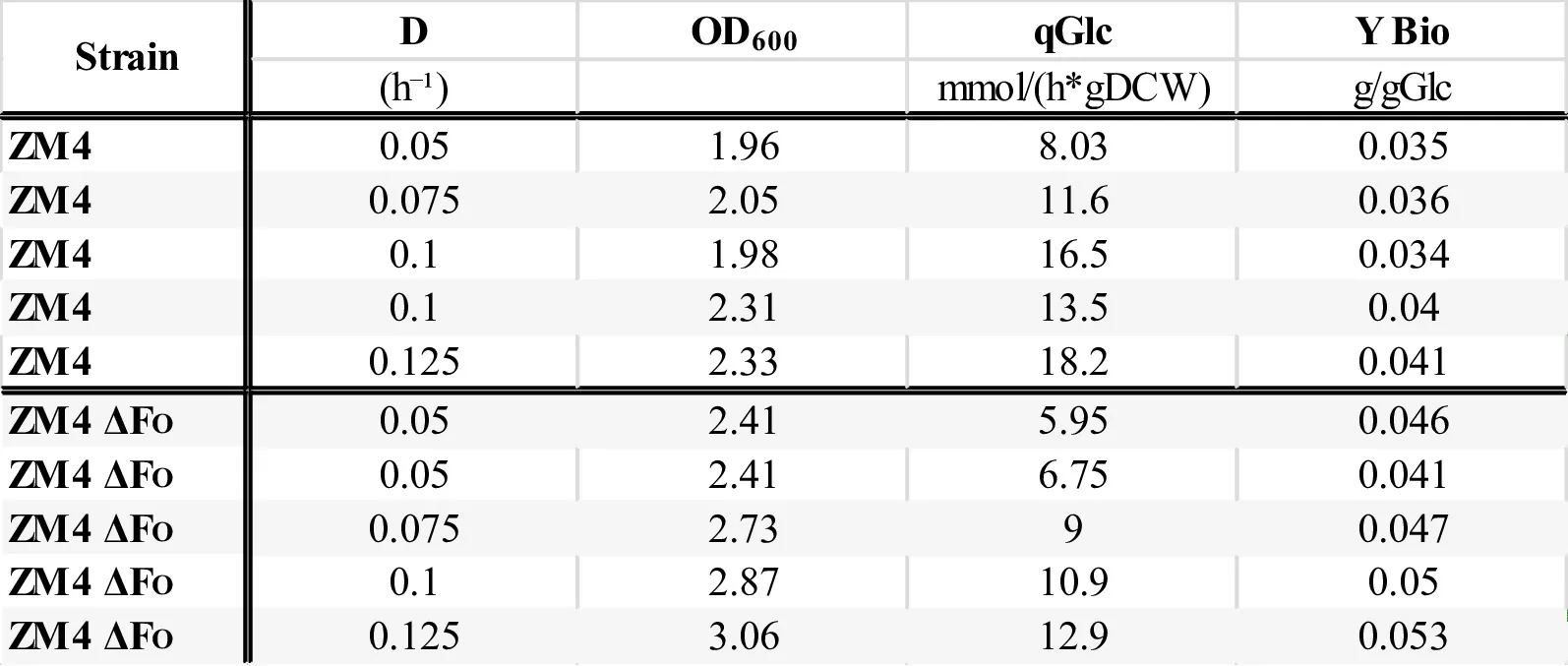
Cell density, glucose consumption rates and biomass yields in glucose-limited chemostat cultivations

### In vitro analysis of the F_O_F_1_ ATPase activity

In vitro studies have shown that the *Z. mobilis* ATPase can operate in the direction of ATP synthesis [40]. To determine the extent of F_O_F_1_-ATPase loss and whether other membrane-associated enzymes compensated for the missing subcomplex activities, we measured the ATPase activity of the membrane fraction in ZM4, ΔF_O_ and ΔF_1_ mutants.

**Fig. 3:**
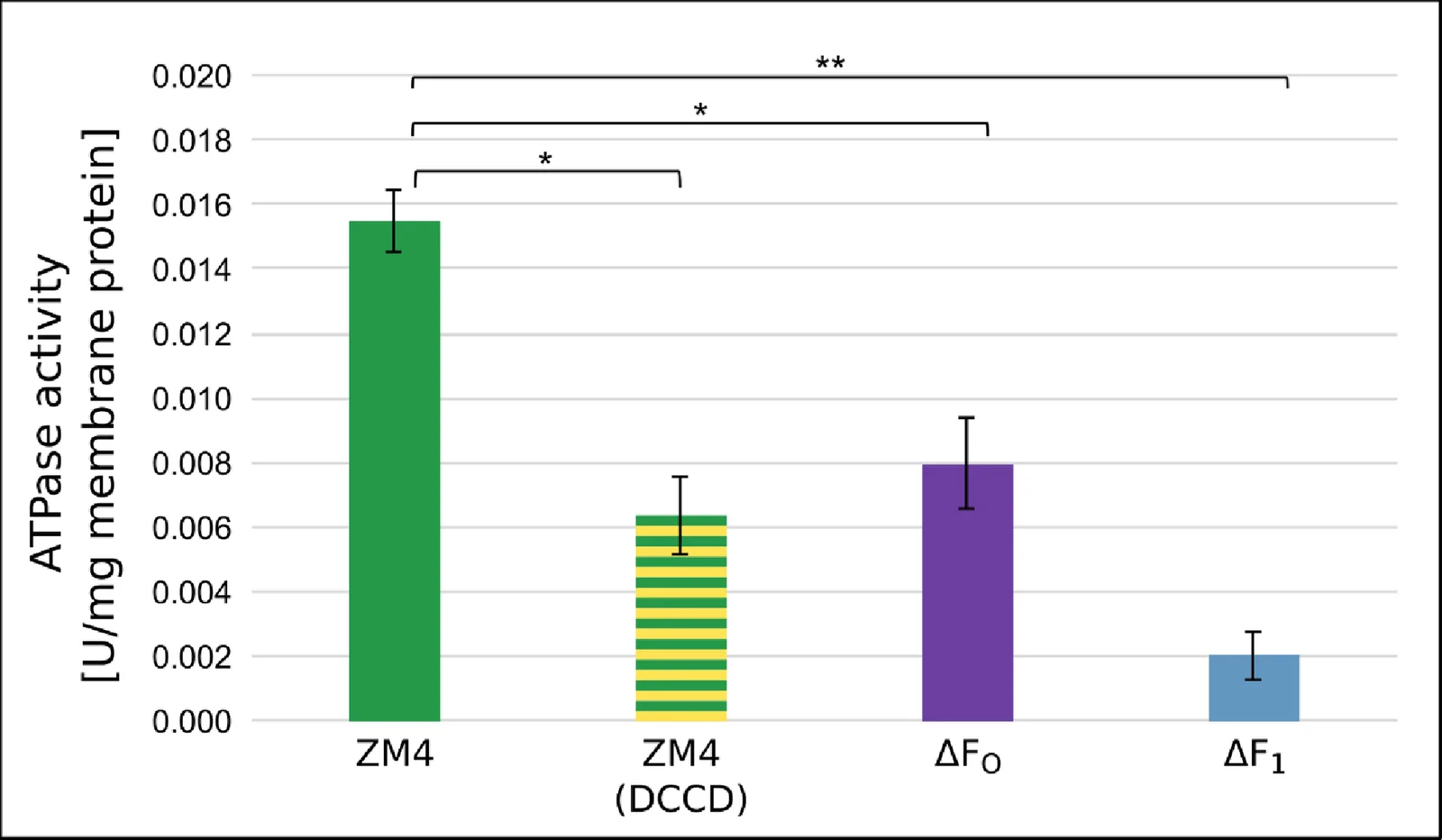
ATPase activity in membrane fractions ZM4, ZM4 with addition of 100µM DCCD, ZM4 ΔF_O_ and ZM4 ΔF_1_ in cell membrane fractions. Assays were performed as described under 3.6. DCCD was added 30 min prior experiment. The figure shows the mean and standard deviation of three independent repeats. Significance of difference between groups was determined via t-test and is indicated as * = *p* < 0.05 and ** *p* < 0.01

Analysis of the membrane fraction ATPase activity clearly showed a significant decrease in both mutant strains compared to the wild-type ZM4 (Fig. 3). The ΔF_1_ strain exhibited an almost complete loss of ATP cleavage ability, confirming the successful functional removal of the catalytic F_1_ subcomplex. However, the ΔF_O_ strain retained detectable ATPase activity, showing a 50% decrease compared to ZM4. This decrease may be due to the presence of the catalytic F_1_ subcomplex still associated with the membrane but uncoupled from the F_O_ proton channel. The residual activity in the ΔF_O_ mutant was comparable to the activity remaining in the wild-type ZM4 membranes after treatment with 100 µM N, N-Dicyclohexylcarbodiimide (DCCD). Since DCCD specifically inhibits the F_O_ proton channel, the partial reduction in ATPase activity observed after DCCD addition confirms that the F_O_F_1_-ATPase in *Z. mobilis* is DCCD-sensitive and confirm that the F1 subcomplex remains active and membrane associated also without a functional F_O_ subcomplex.

**Fig. 4:**
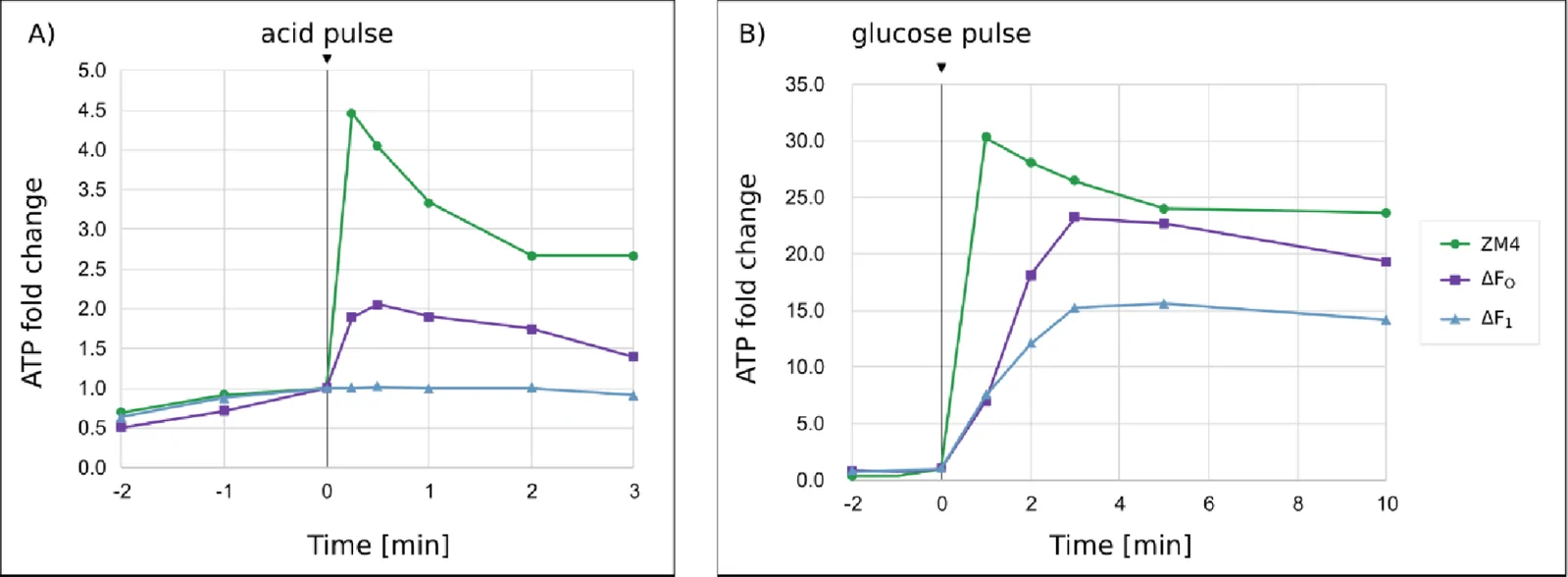
Analysis of ATP synthesis in starved cell suspensions of ZM4 and the mutant strains. **A**) Fold change of intracellular ATP levels of starved cells after acid pulse. **B)** Fold change of intracellular ATP levels of ZM4, ΔF_O_ and ΔF_1_ strains after glucose addition. ATP levels are normalized against the ATP concentration measured at the initiation of the pulse at t = 0.

The results of the ATP synthesis induction experiment using an acid pulse in starved cell suspensions (Fig. 4A) were unexpected yet somewhat aligned with the results our previous experiment (Fig. 3). While starved cells of the ΔF_1_ mutant did not synthesize ATP after an acid pulse, the ΔF_O_ cells exhibited some activity. The increase in the ATP concentration observed for the ΔF_O_ mutant was lower than that determined for ZM4 but clearly observable. One explanation for this result could be that a portion of the F_1_ subcomplex remains associated with the membrane and is active even without the F_O_ subcomplex. However, there is no evidence in the literature of ATP synthase activity in the isolated F_1_ complex. An easy explanation would be the presence of residual F_O_ activity. However, this is very unlikely in our mutant because of the deletion of all genes encoding this subcomplex. One possibility might be that the F_1_ subcomplex finds another interaction partner under these conditions or that the presence of the F_1_ subcomplex stimulates ATP synthase activity by another enzyme. Unfortunately, the reason underlying the measured ATP synthase activity in the ΔF_O_ mutant cannot be concluded from our data.

Starved cells pulsed with glucose can generate ATP via substrate-level phosphorylation, which is independent of the F_O_F_1_ ATPase. A control experiment with a glucose pulse yields similar results to those with an acid pulse (Fig. 4B). Once again, the ΔF_O_ mutant exhibits an activity comparable to that of ZM4 while the activity of the ΔF_1_ cells is low, though not zero.

The reason why cells that have lost the F_1_ subcomplex exhibited decreased substrate-level phosphorylation in this assay is unclear. One possible reason is an uncontrolled flux of H^+^ through the F_O_ pore into the cytoplasm, which leads to acidification of the cytoplasm and the subsequent inactivation of metabolic enzymes in general.

### pH tolerance of ATPase mutant strains

**Fig. 5:**
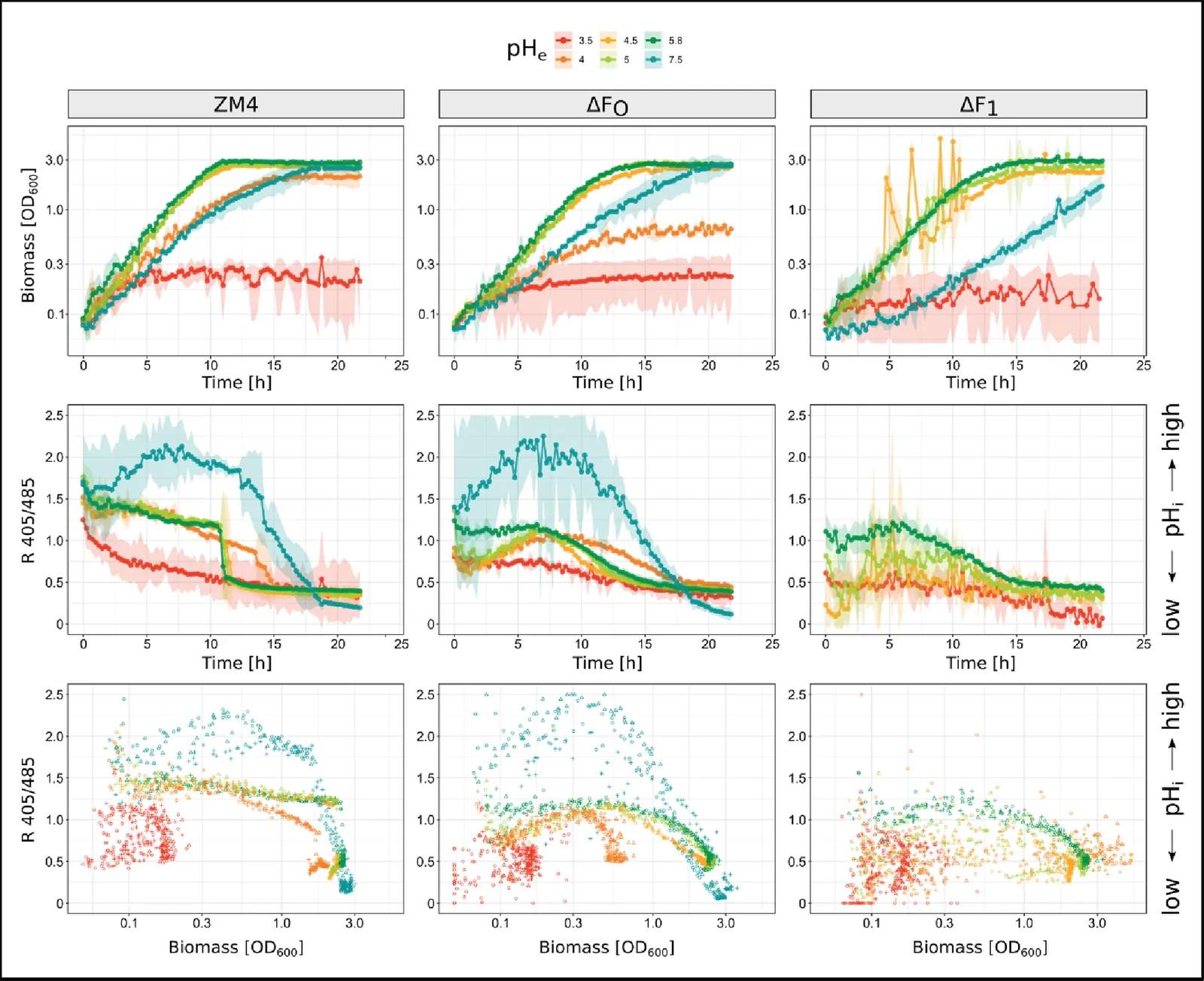
Growth behavior and PHP signals in ZM4, ZM4 ΔF_O_ and ZM4 ΔF_1_ growing in ZM medium with different starting pH_e_ values. Cells from an actively growing preculture were harvested and resuspended in ZM medium with different pH_e_ values. The cultures were inoculated in a microplate reader at 30 °C with vigorous shaking and the OD_600_ was monitored throughout the experiment. All strains carry pZP1573 expressing PHP. Columns are showing data from ZM4 (left), the ΔF_O_ mutant (middle) and the ΔF_1_ mutant (right). The top row shows growth over time via confidence interval (95%). The middle row shows the R 405/485. The bottom row shows the R 405/485 of each replicate (difference in shape indicates different replicates) plotted against the biomass. Shown is either the confidence interval (95%) or the direct measurements, each variation is based on three replicates.

As reported, we observed that both mutant strains did not consume glucose completely in shaking flasks though they were able to do so in bioreactor experiments with constant external pH (pH_e_). Therefore, we suspected that the mutants were more sensitive to lower pH_e_ than the wild type. To analyze the observed effect of low pH_e_ on culture growth more thoroughly, we performed experiments in which the cells were first grown in ZM medium with glucose until reaching mid-exponential phase. Then the cells were harvested and resuspended in ZM medium with different pH ranging from 7.5 to 3.5. These experiments were performed with strains carrying plasmid pZP1573, encoding the ratiometric intracellular pH indicator protein PHP [1]. PHP is a derivative of pHIuorin2 [32] and has been used successfully to monitor intracellular pH (pH_i_) in *Pseudomonas putida* and *E. coli* [1]. To use PHP in *Z. mobilis*, we cloned the coding sequence onto a suitable vector for *Z. mobilis*. Growth assays were carried out in microplates under vigorous shaking and with measuring the OD_600_ and the fluorescence emission at λ ~ 515 nm after excitation at λ ~ 405 nm and λ ~ 485 nm, respectively. Different growth rates were observed for the different strains and the different starting pH_e_ values (Fig. 5).

None of the strains grew in a medium with a starting pH value of 3.5. However, differences could be observed for starting pH values of 4.0. ZM4 grew slightly slower at a starting pH 4.0 than at the control pH levels of 5.0 and 5.8 but it reached a similar cell density at the end of the experiment. In comparison, both mutants were severely impaired at a starting pH value of 4.0. Growth was very slow and only low cell densities were achieved even after prolonged incubation times. In addition to the previously observed differences, cell clumping was observed in the ΔF_1_ strain at a starting pH value of 4.0 and 4.5, resulting in noisy OD_600_ signals (data not shown). Cell clumping of the ΔF_1_ mutant has been observed in our lab before. However, it could not be reconciled with any clear growth condition. We suspect that oxygen supply influences this behavior because clumping often occurred in vigorously shaken flasks but never in the controlled bioreactor experiments. This observation is in agreement with previous studies showing flocculation of *Z. mobilis* strains during aerobic growth in minimal medium and under stress conditions [8, 19].

This growth behavior confirms that the mutants are less tolerant to low pH values than the wild type strain, implying a role of the F_O_F_1_ ATPase in controlling the intracellular pH. In addition to analyzing growth at lower pH levels, we also examined growth of the strains at pH 7.5. A difference between the strains was also apparent for this higher pH. All strains grew more slowly at a starting pH value of 7.5 than at pH 4.0. Growth appears to start with a slight delay in ZM4 and the ΔF_O_ strain, but remains constant afterwards. The delay was much more pronounced in the ΔF_1_ mutant. This strain gradually increased its growth rate throughout the experiment, possibly reflecting a decrease in medium pH during growth.

To investigate how the external pH_e_ influences the intracellular pH of the mutant strains we used the pH-sensitive fluorescence reporter PHP (pH indicator for Pseudomonas), a variant of pHluorin2 [1, 32] to estimate the internal pH of the strains. The bimodal excitation spectrum of pHluorin2 is pH dependent; the ratio of the fluorescence intensities at λ_Em_ = 515 nm after excitation at λ_Ex_ = 405 nm and λ_Ex_ = 485 nm (R405/485), respectively, can be used to infer the pH of the environment of pHluorin2 [1]. A high R405/485 ratio indicates a high pH while a low ratio indicates a low pH.

Using pHluorin2 enabled us to investigate pH_i_ noninvasively over the entire growth period. The calibration curve that assigns a pH_i_ value to an R405/485 ratio can only be used to a limited extent to calculate the actual pH_i_ because not all the values acquired from the growth curves did fall within the same R405/485 scale (Suppl Fig. 4). We suppose that this is due to the influence of buffering, media or protein concentrations or another uncontrollable factor in our in vitro data. Nevertheless, the R405/485 ratio should still provide a qualitative picture of the changes in pH_i_. Under favorable growth conditions (with a starting pH_e_ of 4.5, 5.0 and 5.8) the wild-type strain exhibits two rather distinct R405/485 signals: an R405/485 ratio of 1.5 to 1.2 during growth and an R405/485 ratio of 0.5 to 0.3 during stationary phase (Fig. 5). The transition is rapid, indicating a pH_i_ shift from 7 to 7.5 to around 4, assuming that the calibration curve is accurate. The intracellular pH of *Z. mobilis* has been reported to be low (around pH 5.5–6.5) but as in our results to be varying in response to growth conditions [20, 34, 35].

Under unfavorable growth conditions these modes are still apparent, however, the mutant strains exhibit slightly different behavior, with the pH_i_ dropping more gradually. Additionally, both mutant strains exhibit lower R405/485 ratios during their growth phase.

The wild type strain ZM4 exhibits a less pronounced decrease in pH_i_ during active growth than the mutants and appears capable of maintaining a higher pH_i_ at higher biomass concentrations than the mutant strains. Since both mutant strains grow more slowly, their growth period is also longer and the drop in pH_i_ occurs at a later time point and at a lower biomass concentration than in the wild type. The plot of R405/485 against OD_600_ corrects for the different growth speeds (see Fig. 5 bottom row).

Interestingly, prior observations of the pH_i_ of an ATPase mutant strain of *Corynebacterium glutamicum* showed no difference in pH_i_ [25]. In that study, however, the pH_i_ was only determined at one time point during a cultivation with fixed external pH. Additionally, they used a complete ATPase knockout strain. Our results suggest that both ATPase subcomplexes, F_O_ and F_1,_ are involved in the pH_i_ maintenance in *Z. mobilis*. Based on our data it is tempting to speculate that during growth protons must be pumped out of the cell in order to reduce the intracellular proton concentration and prevent acidification of the cytoplasm. In wild-type cells ATPase performs this action. Furthermore, Enright et al. [12] have studied the membrane energization by *Z. mobilis* ATPase using partial ATPase knock-down strains. They presented evidence that membrane energization by ATPase drives the electron transfer from NADH to ferredoxin by Rnf electron transport complex, supplying reducing equivalents for isoprenoid biosynthesis pathway in anaerobically growing cells. In the mutants at least part of the ATPase function can be fulfilled by the electron transport chain, which explains the oxygen demand of the mutants.

## Conclusions

We analyzed *Z. mobilis* ZM4 mutants with deletions in the F_O_ and the F_1_ subcomplexes of the ATPase. Our data indicate that both subunits of the F_O_F_1_ ATPase are essential for anaerobic growth in *Z. mobilis*. Our results suggest that the primary function of the ATPase of *Z. mobilis* is to establish a proton gradient across the membrane or pump protons out of the cytoplasm, which is coupled to ATP cleavage. The proton gradient is essential for growth and is typically associated with important functions such as nutrient uptake, intracellular pH homeostasis and motility. Similarly, it is important for cells to maintain an intracellular pH favorable for growth. Under aerobic conditions, the respiratory chain can also build up a proton gradient and take over part of the ATPase function, allowing the ΔF_O_ and ΔF_1_ mutants to grow. Our data also suggest that a significant amount of ATP is used in this process and that the high ATP turnover by the ATPase is linked to the high glucose uptake rate of ZM4. This is evident from the reduced glucose uptake rates of both mutants. Therefore, the uncoupled growth phenotype of *Z. mobilis* seems to be largely caused by the proton pumping activity of its F_O_F_1_ ATPase.

## Supplementary Information

Below is the link to the electronic supplementary material.

Supplementary Material 1.

## Data Availability

All data supporting the findings of this study are available within the paper and its Supplementary Information. Sequence information can be found in an Edmond repository under https://doi.org/10.17617/3.X1LFHY.
