# Supplementary material for: Investigating the role of FOF1 ATPase in Zymomonas mobilis through deletion of its FO and F1 subcomplexes
Source: Microb Cell Fact. 2026 Jul 13;25:161. doi: 10.1186/s12934-026-03059-x (PMC13366798; doi:10.1186/s12934-026-03059-x)
Supplement: Supplementary file 1 — Supplementary Material 1. [file 12934_2026_3059_MOESM1_ESM.docx]

**Supplementary File 1**

**Investigating the role of F_O_F_1_ ATP Synthase in *Zymomonas mobilis* Through Deletion of Its F_O_ and F_1_ subunits**

Gerrich Behrendt^1^, Reinis Rutkis^2^, Uldis Kalnenieks^2^, Katja Bettenbrock^1,^*

1) Max-Planck-Institute for Dynamics of Complex Technical Systems, Sandtorstraße 1, 39106 Magdeburg, Germany

2) Institute of Microbiology and Biotechnology, University of Latvia, 1 Jelgavas Str., LV-1004 Riga

*) Corresponding author: bettenbrock@mpi-magdeburg.mpg.de

**Suppl. Table 1: Plasmids used in this work.**

| **Plasmid name** | **Description** | **Assembly** | **Literature source** |
| --- | --- | --- | --- |
| pUC19-dBsaI-dSmaI | pUC19-ΔBsaI-ΔSmaI | Found in reference. | [1] |
| pGB-MPI-003 | ColE1-pZMOB06-knR | Found in reference. | [1] |
| pZP001 | acceptor-lvl0-pos001 | Found in reference. | [1] |
| pZP003v2 | acceptor-lvl0-pos003v2 | Found in reference. | [1] |
| pZP013B | acceptor-lvl1-pos1-crDNA | Primer 48 + Primer 49 (Templ. crDNA-Acceptor_cloned_in_pZP001, Atemp. 60 °C, 1300 bp) SmaI Cut-Ligation in pGB-MPI-003 | [2] |
| pZP014 | acceptor-lvl1-pos2 | Found in reference. | [1] |
| pZP016 | acceptor-lvl1-pos4 | Found in reference. | [1] |
| pZP022 | acceptor-lvl1-pos3-reverse | Found in reference. | [1] |
| pZP037 | lvl2-end-linker-4 | Found in reference. | [1] |
| pZP063 | lvl1-pos1-crDNA-ZMO0668-Fo | Primer 136 + Primer 137 annealed BsaI Cut-Ligation pZP013B | This article. |
| pZP064 | lvl1-pos4-dsHA-ATPase-Fo-cluster | Primer 138 + Primer 139 (Templ. ZM4 gDNA, Atemp. 65 °C, 800 bp) BsaI Cut-Ligation in pZP016 | This article. |
| pZP065 | lvl1-pos2-usHA-ATPase-Fo-cluster | Primer 140 + Primer 141 (Templ. ZM4 gDNA, Atemp. 65 °C, 600 bp), Primer 142 + Primer 143 (Templ. ZM4 gDNA, Atemp. 65 °C, 100 bp), Primer 144 + Primer 145 (Templ. ZM4 gDNA, Atemp. 65 °C, 108 bp) BsaI Cut-Ligation in pZP014 | This article. |
| pZP067 | lvl1-pos1-crDNA-ZMO0240-F1 | Primer 146 + Primer 147 annealed BsaI Cut-Ligation pZP013B | This article. |
| pZP068 | lvl1-pos2-usHA-ATPase-F1-cluster | Primer 148 + Primer 149 (Templ. ZM4 gDNA, Atemp. 65 °C, 800 bp) BsaI Cut-Ligation in pZP014 | This article. |
| pZP069 | lvl1-pos4-dsHA-ATPase-F1-cluster | Primer 150 + Primer 151 (Templ. ZM4 gDNA, Atemp. 65 °C, 800 bp) BsaI Cut-Ligation in pZP016 | This article. |
| pZP131 | lvl1-pos3rev-EU-selection-marker-spR | Primer 326 + Primer 327 (Templ. pEZ15A-Dual, Atemp. 60 °C, 1000 bp) BsaI Cut-Ligation in pZP022 | This article. |
| pZP132 | lvl1-pos3rev-EU-selection-marker-knR | Found in reference. | [1] |
| pZP137 | acceptor-lvl2-pos1-ZM-suicide | Primer 74 + Primer 75 (Templ. pUC19-dBsaI-dSmaI, Atemp. 65 °C, 600 bp) SmaI Cut-Ligation in pUC19-dBsaI-dlacZa | This article. |
| pZP159 | lvl0-pos-002v2-ZM4-RBS-10k | Found in reference. | [1] |
| pZP289 | lvl0-pos004-terminator-of-soxR | Found in reference. | [1] |
| pZP337 | lvl2-editing-ATPase-Fo-cluster-KO-knR-KI-suicide | pZP137 + pZP063 + pZP064 + pZP065 + pZP132 + pZP037 BbsI Cut-Ligation | This article. |
| pZP338 | lvl2-editing-ATPase-F1-cluster-KO-spR-KI-suicide | pZP137 + pZP067 + pZP068 + pZP069 + pZP131 + pZP037 BbsI Cut-Ligation | This article. |
| pZP436* | lvl0-pos001-Pstrong100k | Found in reference. | [1] |
| pZP992 | lvl0-pos003v2-pHluorin2 | Primer 916 + Primer 917 (Templ. pS2513-PHP , Atemp. 60 °C, 700 bp) BbsI Cut-Ligation in pZP003v2 | This article, [3] |
| pZP1007 | acceptor-lvl1-pos1-mob-cmR-pZMOB06 | Found in reference. | [1] |
| pZP1573 | lvl1-pos1-mob-ori-pZMOB06-cmR-Pstrong100k-pHluorin2-TsoxR | pZP1007 + pZP436* + pZP159 + pZP992 + pZP289 BsaI Cut-Ligation | This article. |

**Suppl Table 2: Primer Sequences**

| **Primer** | **Sequence (5´...3´)** |
| --- | --- |
| Primer 48 | ggaGAAGACaaTGCCAATATAAGGTCGCTCTTTTGAAGAGCGG |
| Primer 49 | ggaGAAGACaaTTGCTCCCTAATAAAAAACAAACCGTTTTTTCAATAAAAATCTTAAAAAAAG |
| Primer 74 | ggaGGTCTCaAGTGaaTGCCaaGTCTTCCGCGTTGGCCGATTCATTAATGCAGC |
| Primer 75 | ggaGGTCTCaCCTGaaTCCTaaGTCTTCTCAGGGCGCGTCAGCGGG |
| Primer 136 | GAAAGATGGCCAGCAGGGCCGTTTGTTCATCGGTTT |
| Primer 137 | GAACAAACCGATGAACAAACGGCCCTGCTGGCCATC |
| Primer 138 | ggaGGTCTCaAGTGTAGGATTGTTATGTCTGTACAAATCGGCGTCGTCG |
| Primer 139 | ggaGGTCTCaGGTTGGCGCTTACGATTGCGGAAGAAACTATCAGAG |
| Primer 140 | ggaGGTCTCaAGTGCCAGCTTGCTGATGGTAGGGGGC |
| Primer 141 | ggaGGTCTCaAGCAATTCAGCAGAATAACGCCTATTTACAGGTAAAAATCCCTTCCA |
| Primer 142 | ggaGGTCTCaTGCTCTCTTTTTGCGATGGGAATAGTAGAGAAGTTTGGTTTTCC |
| Primer 143 | ggaGGTCTCaGTAGACGGGTGAAGCAAGTCAACTCAAAGCAC |
| Primer 144 | ggaGGTCTCaCTACTTAAAAAAGACCTACTTGACGAGATAGTTACTCGTCATGCTGAAT |
| Primer 145 | ggaGGTCTCaGGTTCGCCAACTCGCTACGCCTTGA |
| Primer 146 | GAAAGGTTACTTTCGAAAATGCGCAGAAAATCGCTC |
| Primer 147 | GAACGAGCGATTTTCTGCGCATTTTCGAAAGTAACC |
| Primer 148 | ggaGGTCTCaAGTGACATAAGCCGGATGGCGTTCTGATTATCTCG |
| Primer 149 | ggaGGTCTCaGGTTGCGCCTGCCGTCCCC |
| Primer 150 | ggaGGTCTCaAGTGTTCTATTAGCTATCTTCTTTTCAGCCTAAATAAAAACAGATTGAGCGACT |
| Primer 151 | ggaGGTCTCaGGTTCTTGGCTTTAATGTAGCCCCACCCCAACA |
| Primer 326 | ggaGGTCTCaAGTGcacctgaagtcagccccatacg |
| Primer 327 | ggaGGTCTCaGGTTcagctctctaacgcttgagttaagcc |
| Primer 916 | ggaGAAGACgcAatgagcaaaggtgaagaattgttcacc |
| Primer 917 | ggaGAAGACaaTTCGttacttgtagagttcatccataccgtgg |


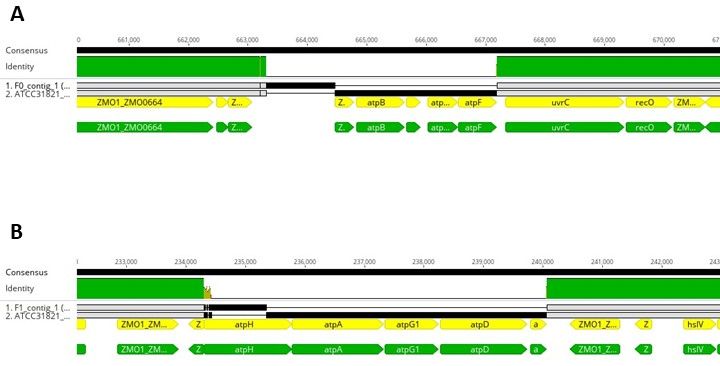


**Suppl Fig. 1: Validation of the mutant strains.**

The mutant strains ZM4ΔF_O_ and ZM4ΔF_1_ were validated by genome sequencing. The respective regions of a chromosome alignment of the genome sequences obtained against the genome of ATTC31821 are shown. Panel A) shows the region of the ΔF_O_ mutant and B) shows the region of the ΔF_1_ mutant. Green identity boxes indicate sequence identity. Black boxes indicate sequences that are present in only one of the aligned genomes. As can be seen for both mutants, the genes encoding the respective ATPase subunits are absent, and an additional sequence corresponding to the antibiotic resistance cassette has been inserted

Chromosomal DNA of the mutants was prepared using the kit (NEB). Sequencing was performed using the Oxford Nanopore sequencing service from Eurofins Genomics. The obtained sequences were aligned using the Mauve plugin in Geneious Prime software.


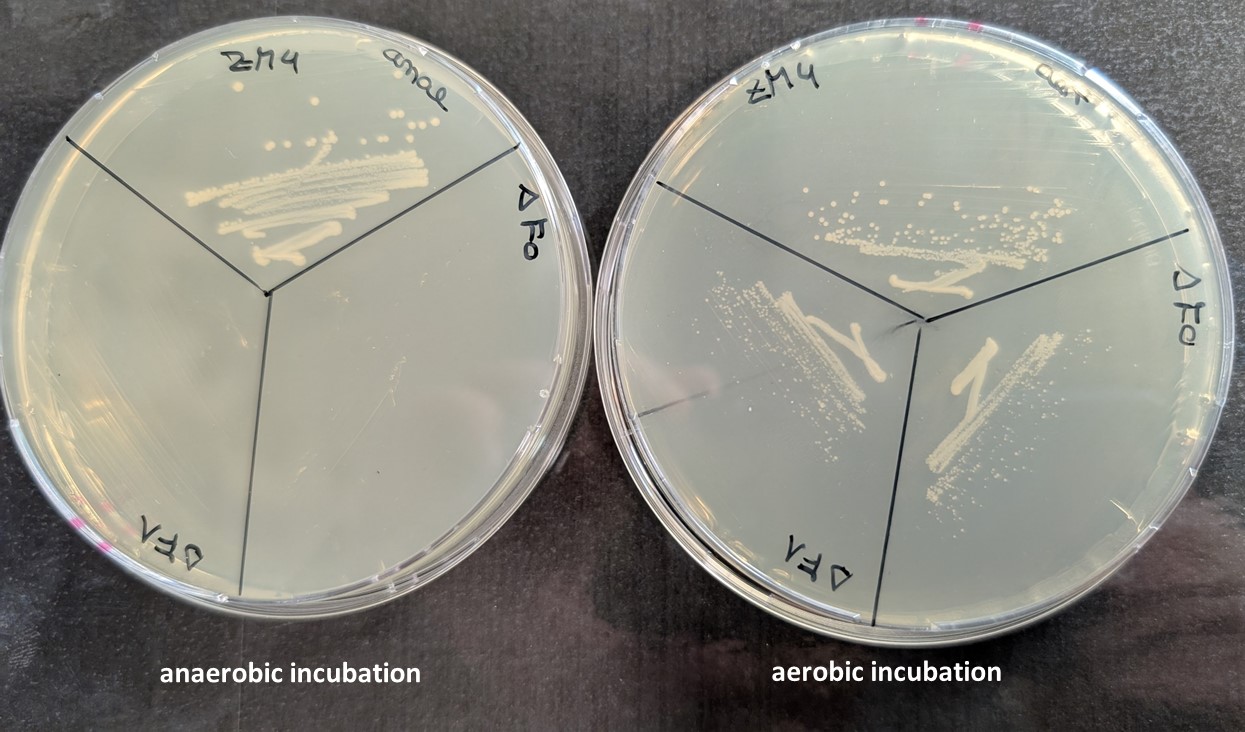


**Suppl Fig 2: Growth of ZM4 and the ΔF_O_ and ΔF_1_ mutants of ZMGlc plates incubated under anaerobic or aerobic condtitions.** The plates were incubated at 30°C for 48 hours. ZM4 showed good growth under both conditions, whereas the mutant strains showed no growth under anaerobic conditions.


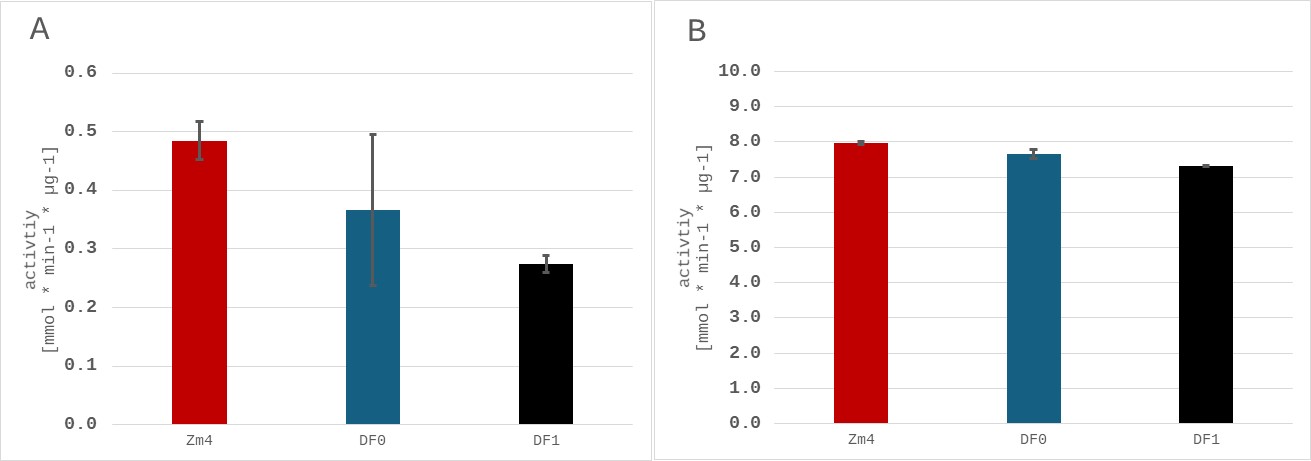


**Suppl Fig. 3. NADH:Q1 oxidoreductase activity in the membrane fractions of ZM4 and the the ΔFO and ΔF1 mutants.** Shown are the the activites using A) NADH and B) Q1 as electron donor.

| 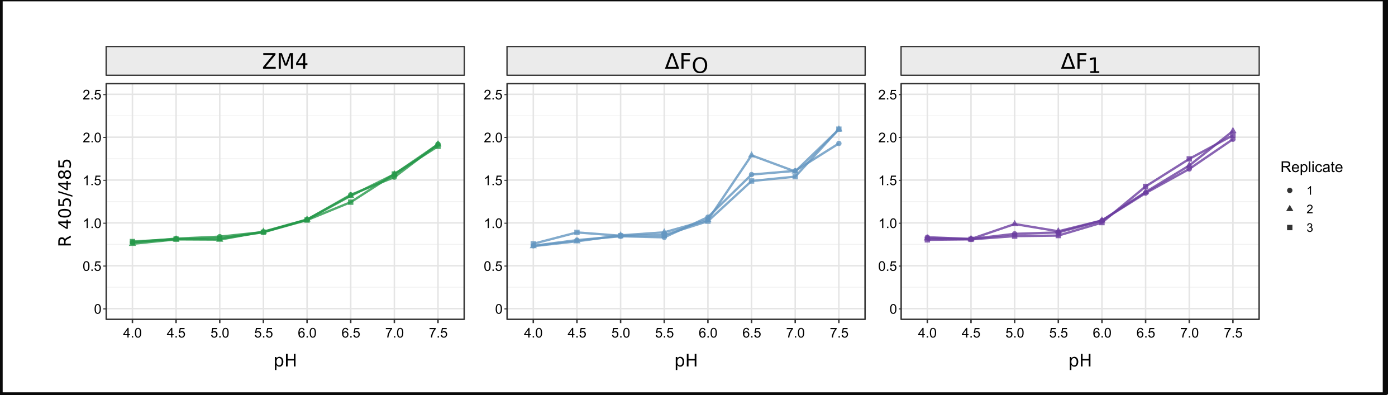 |
| --- |

**Supplementary Fig. 4: Calibration curves of PHIuorin2 signals of ZM4, ZM4 ΔFO and ZM4 ΔF1 in ZM medium with different pH values.** Calibaration war performed as described by (Arce-Rodríguez et al., 2019). Cells from actively growing precultures were harvested and resuspended in ZM medium with 50 mM sodium benzoate and 50 mM methylamine, set to different pH values. Fluorescence measurements were carried out in a microplate reader (Vantastar, BMG Labtec) at 30 °C. All strains carried pZP1573 expressing pHluorin2.
